# Effects of acetylcholine on neuronal properties in entorhinal cortex

**DOI:** 10.3389/fnbeh.2012.00032

**Published:** 2012-07-24

**Authors:** James G. Heys, Nathan W. Schultheiss, Christopher F. Shay, Yusuke Tsuno, Michael E. Hasselmo

**Affiliations:** Graduate Program for Neuroscience, Center for Memory and Brain, Boston UniversityBoston, MA, USA

**Keywords:** entorhinal cortex, spatial navigation, oscillatory interference

## Abstract

The entorhinal cortex (EC) receives prominent cholinergic innervation from the medial septum and the vertical limb of the diagonal band of Broca (MSDB). To understand how cholinergic neurotransmission can modulate behavior, research has been directed toward identification of the specific cellular mechanisms in EC that can be modulated through cholinergic activity. This review focuses on intrinsic cellular properties of neurons in EC that may underlie functions such as working memory, spatial processing, and episodic memory. In particular, the study of stellate cells (SCs) in medial entorhinal has resulted in discovery of correlations between physiological properties of these neurons and properties of the unique spatial representation that is demonstrated through unit recordings of neurons in medial entorhinal cortex (mEC) from awake-behaving animals. A separate line of investigation has demonstrated persistent firing behavior among neurons in EC that is enhanced by cholinergic activity and could underlie working memory. There is also evidence that acetylcholine plays a role in modulation of synaptic transmission that could also enhance mnemonic function in EC. Finally, the local circuits of EC demonstrate a variety of interneuron physiology, which is also subject to cholinergic modulation. Together these effects alter the dynamics of EC to underlie the functional role of acetylcholine in memory.

## Introduction

There is strong evidence to support the claim that acetylcholine modulates the physiology and the function of the entorhinal cortex (EC). From behavioral experiments in humans, non-human primates and rodents it is clear that cholinergic activity can affect performance in memory tasks (for review see: Hasselmo, 2006; Deiana et al., 2011; Newman et al., 2012). From *in vivo* electrophysiological recordings in awake-behaving animals and electrophysiological recordings using *in vitro* slice preparations it is clear that acetylcholine can modulate many aspects of the neurophysiology in EC. The purpose of this review will be to summarize data, primarily from the rat, that demonstrates how acetylcholine activity can modulate the physiology of EC, paying specific attention to cellular mechanisms that may underlie cholinergic dependent modulation of EC function. This paper can be read independently or in concert with Newman et al. (2012) in the same issue, which provides a further description of the behavioral correlates of cholinergic modulation that relate to the function of EC.

This review is broken into four major parts. The first section provides an overview of the anatomy and the basic physiology of EC and the major cholinergic input to the EC, the medial septum, and vertical limb of the Diagonal Band of Broca (MSDB). The second section focuses upon cholinergic modulation of subthreshold electrophysiological properties of neurons in medial entorhinal cortex (mEC). The focus of the third section is upon suprathreshold physiology, including a description of the role of acetylcholine in persistent spiking activity, ionic mechanisms that likely underlie this unique firing activity and cholinergic modulation of synaptic transmission in the EC. Finally, the last section focuses upon modulation of interneurons, which have been shown to exhibit substantial diversity in other brain regions and therefore have been detailed in a separate section.

## Anatomy and cytoarchitecture of the basal forebrain and entorhinal cortex

**Figure 1:**
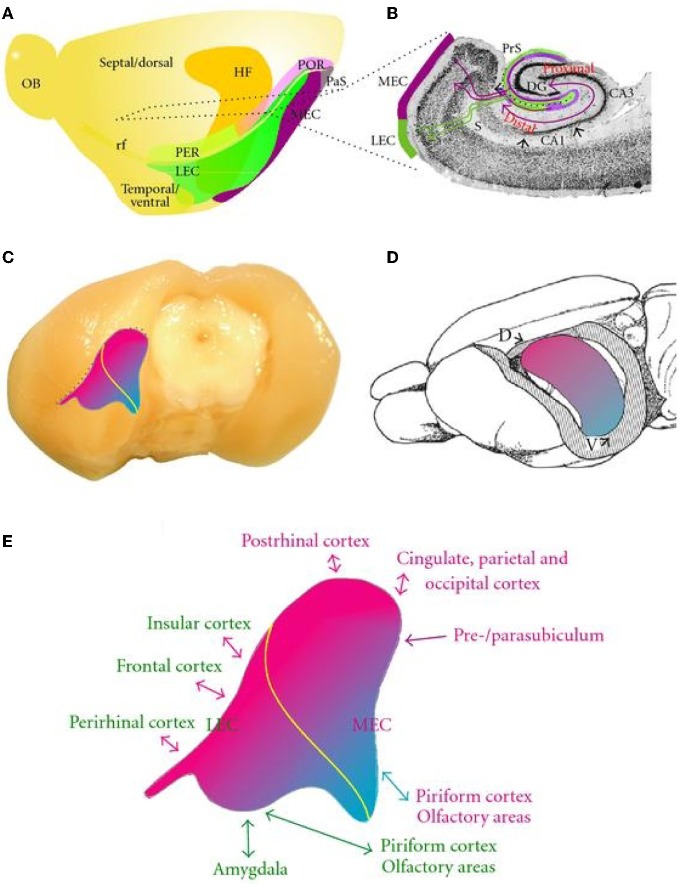
**Schematic representation of the overall organization of the entorhinal cortex and its connectivity. (A)** Position of the entorhinal cortex and surrounding cortices and hippocampus in the rat left hemisphere. Indicated are the dorsoventral extent of the hippocampus, positions of LEC and MEC, and the approximate position of a representative horizontal section, illustrated in **(B)**. **(B)** Horizontal section illustrating entorhinal-hippocampal connectivity (see text for more details). **(C)** and **(D)** Representation of the topographical arrangement of entorhinal-hippocampal reciprocal connections. A dorsolateral band of entorhinal cortex (magenta) is preferentially connected to the dorsal hippocampus. Increasingly, more ventral and medial bands of entorhinal cortex (purple to blue) are connected to increasingly more ventral levels of the hippocampus. Yellow line in **(C)** indicates the border between LEC and MEC. **(E)** Enlarged entorhinal cortex, taken from **(C)**, indicating the main connectivity of different portions of entorhinal cortex. Brain areas preferentially connected to LEC are printed in green, those connected to MEC are in magenta. The color of the arrows indicates preferential connectivity to the dorsolateral-to ventromedial bands of entorhinal cortex (magenta or blue, respectively) or that no preferential gradient is present (green) (taken with permission from Canto et al., 2008).

In the rodent brain, the most common anatomical characterization of the EC is to divide the area into a medial and a lateral subdivision, which is delineated based upon differential connectivity with the dentate gyrus. The mEC projects to the middle one-third of the molecular layer of dentate gyrus whereas the lateral entorhinal cortex (lEC) projects to the outer one-third (Figure 1) (Steward, 1976; Van Groen et al., 1993). Other studies have divided the EC into three distinct rostro-caudal bands based upon exclusive projections to different positions along the longitudinal axis of the dentate gyrus (Dolorfo and Amaral, 1998).

The EC is a six layer structure with perikaryon of principal cells located in layers II, III, V, and VI (Lorente de Nó, 1933; for review see Wouterlood, 2002). In both mEC and lEC, layer I is a molecular layer which contains the distal dendrites of principal neurons in the superficial and deep layers. Layers II and III in mEC and lEC both contain medium to large principal cells. Layer II in mEC can be differentiated from layer II of lEC due to the high density of glutamatergic, large spiny stellate cells (SCs) present exclusively in layer II of mEC (Klink and Alonso, 1993), whereas layer II of lEC contains fan cells with similar morphology to SCs, but have significantly different physiological profiles (Alonso and Klink, 1993; Tahvildari and Alonso, 2005; Canto and Witter, 2011; Shay et al., 2012). Layer III of mEC and lEC contains predominately loosely packed, large to medium pyramidal neurons (Steward, 1976; Steward and Scoville, 1976; Witter and Groenewegen, 1984). The spiny dendritic arbor of SCs radiates in all directions, extending into layer I and across layer II. The deeper layers V and VI have a less recognizable interlaminar border, but can be easily differentiated from the superficial layers due to an empty cell layer IV which is referred to as lamina dissecans. Principal neurons in layer V and VI can be roughly described as pyramidal, horizontal, or polymorphic-based upon dendritic arborization (Hamam et al., 2000, 2002).

The medial septum and vertical limb of the diagonal band of Broca provide the major cholinergic input to the EC (Beckstead, 1978; Alonso and Köhler, 1984; Insausti et al., 1987). Although there are neurons in the MSDB that express a range of classical neurotransmitters and neurohormones, this review focuses on the population of putative cholinergic neurons expressing choline acetyltransferase (ChAT) and the separate population of GABA-ergic neurons expressing GAD, which together comprise the main MSDB projection to the EC (Kiss et al., 1990; Gritti et al., 1993; Manns et al., 2001). Cells in both the GABAergic and cholinergic populations range in size and shape from bipolar to multipolar with somata ranging in size from small (10–15 μm diameter) to large (30–40 μm diameter) (Gritti et al., 1993; Manns et al., 2001). Furthermore, the firing properties of the two populations show no clear differences, making it difficult to define their functional roles (Simon et al., 2006).

While there has been some research directed toward understanding the reciprocal connectivity between the basal forebrain and the EC, much of the knowledge of this pathway has come through studies that have been primarily focused upon forebrain-hippocampal projections (Jones et al., 1976; Meibach and Siegel, 1977; Mesulan et al., 1983; Milner et al., 1983; Amaral and Kurz, 1985; Nyakas et al., 1987; Kiss et al., 1990; Gaykema et al., 1991). It is important to note that in the rat, the connectivity patterns that exist between the basal forebrain and the hippocampus differ considerably from those of the basal forebrain and neocortex. In particular, the hippocampus receives the majority of its basal forebrain projections from the MSDB, whereas the neocortex receives its forebrain cholinergic input from the nucleus basalis of Meynert (Jones et al., 1976; Mesulan et al., 1983; Amaral and Kurz, 1985). As the laminar structure of the EC can be viewed as a developmental middle ground between the tri-layered hippocampus and six layered neocortex, careful attention must be paid when using anatomical data of forebrain projections to neocortex or the hippocampus to make predictions about entorhinal-basal forebrain connectivity.

Anatomical tracing studies have demonstrated several topographic patterns along the axis of the MSDB. When studied as a whole, MSDB fibers projecting to the hippocampus maintain rostral-caudal specificity such that neurons located rostrally in the MSDB project to septal levels of the hippocampus (Meibach and Siegel, 1977; Milner et al., 1983; Amaral and Kurz, 1985). This pattern is in contrast to the topography of cholinergic neurons in the MSDB, which seem to follow an inverse projection pattern. ChAT positive neurons in the dorsal MSDB send axons preferentially to the temporal pole of the hippocampus, whereas ChAT positive neurons in the ventral band of the MSDB send a higher percentage of their output to septal hippocampus (Amaral and Kurz, 1985). There is also data to suggest that there are relatively more cholinergic neurons throughout the MSDB that project to temporal levels of the hippocampus (Hoover et al., 1978; Milner et al., 1983; Amaral and Kurz, 1985). This could arise from the mediolateral topographic organization that is observed, especially within the dorsal band of MSDB. Increased ChAT expression is seen in the lateral aspect of the dorsal band of MSDB, which project preferentially to temporal hippocampus (Meibach and Siegel, 1977; Amaral and Kurz, 1985; Nyakas et al., 1987; Kiss et al., 1990; Gaykema et al., 1991). This is contrast to the medial aspect of the dorsal band of MSDB, which show lower levels of ChAT expression and project more strongly to the septal pole of the hippocampus.

Early anatomical tracing studies focusing on the EC of the rat demonstrated that the mEC and lEC receive overlapping, yet distinct patterns of input from the MSDB. While neurons in the MSDB send projections throughout the extent of the EC, the diagonal band of Broca projects preferentially to the lEC, and this is contrast to the medial septal projection which targets the mEC (Beckstead, 1978; Insausti et al., 1987; Kerr et al., 2007). Future studies are needed to determine whether similar topographic projection patterns exist from the MSDB to the entorhinal cortex as seen in the projections of the MSDB to the hippocampus. In particular, a similar topography of MSDB projections along the septal-temporal axis of the hippocampus may exist in MSDB efferents along the dorsal-ventral axis of EC and this could result in differential cholinergic modulation at different positions along the dorsal-ventral axis of the EC. These potential anatomical and physiological features have important implications for mEC where the spatial representation of the neurons in mEC grid cells changes in neurons along the dorsal to ventral axis (Fyhn et al., 2004; Hafting et al., 2005).

## Overview of EC physiology

**Figure 2:**
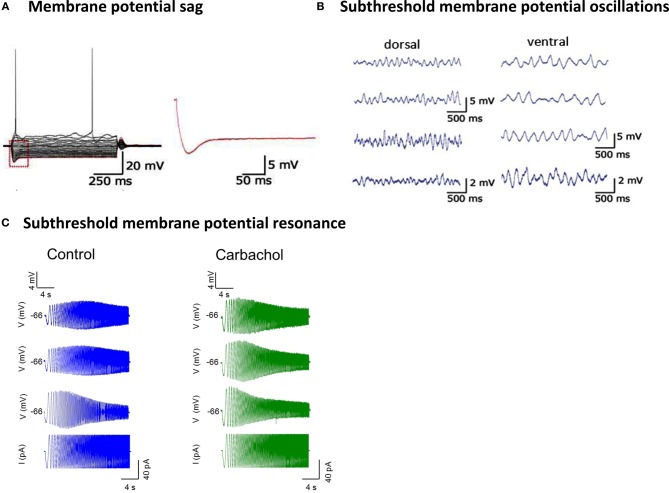
**Subthreshold electrophysiological properties of stellate cells in medial entorhinal cortex. (A)** Whole cell patch clamp recordings demonstrate that hyperpolarizing current injections from a membrane potential of approximately −60 mV produces membrane potential sag in SCs. Inset: the sag amplitude is measured as the difference between the hyperpolarized membrane potential and the steady state membrane potential. **(B)** Subthreshold membrane potential oscillations recorded in dorsal stellate cells (left) and ventral stellate cells (right) at average membrane potentials of −50 mV (top) and −45 mV (bottom). **(C)** Subthreshold membrane potential resonance characterized in current clamp recordings of three different stellate cells. The membrane potential (top three traces) is shown response to the sinusoidal current injection (bottom trace). The subthreshold membrane resonance is measured in control (blue) and after application of carbachol (green). (Figures 2A and 2B reprinted with permission from Giocomo et al., 2007. Figure 2C reprinted with permission from Heys et al., 2010).

The superficial layers of EC contain two general classes of principal neurons. Both layers II and III contain regular-spiking pyramidal neurons, whereas layer II also contains large excitatory SCs in mEC and “fan cells” with similar morphology in lEC (Alonso and Llinás, 1989; Alonso and Klink, 1993; Klink and Alonso, 1993; Jones, 1994; Dickson et al., 1997). SCs in mEC can be distinguished electrophysiologically due to the presence of a large amplitude hyperpolarization activated cation current (I_h_) (Klink and Alonso, 1993; Dickson et al., 2000a,b). Using whole-cell patch clamp recordings in current clamp, I_h_ manifests in the characteristic “sag” response due to hyperpolarizing current injection (Figure 2A). While there have not been comprehensive voltage clamp studies to isolate and directly compare I_h_ across all known principal cells in the EC, a reasonable estimate of I_h_ using the sag ratio suggests that I_h_ present in SCs is at least two times larger than any other class of principal cells that has been recorded throughout the EC (Alonso and Klink, 1993; Dickson et al., 1997, 2000a,b; Hamam et al., 2000, 2002; Tahvildari and Alonso, 2005). In addition, layer II SCs show large amplitude subthreshold membrane potential oscillations at theta frequencies (4–12 Hz) (Figure 2B) and subthreshold membrane potential resonance at theta frequencies (4–12 Hz) (Figure 2C) (Alonso and Llinás, 1989; Erchova et al., 2004; Giocomo et al., 2007; Heys et al., 2010; Shay et al., 2012). Interestingly, all three electrophysiological features of SCs change systematically along the dorsal to ventral axis of mEC (Giocomo et al., 2007), which coincides with systematic changes in the characteristic spatial representation of grid cells along the same anatomical axis of mEC (Hafting et al., 2005). The subthreshold membrane potential oscillations have an average frequency of 6.42 ± 0.40 Hz in dorsal SCs at −50 mV (Figure 2B, left) and 4.23 ± 0.32 Hz in ventral SCs at −50 mV (Figure 2B, right).

The electrophysiological properties of neurons in the deep layers of EC have been studied by several groups. However, the three morphological classes of principal neurons (pyramidal, horizontal, or polymorphic) do not seem to display any distinguishing electrophysiological features (Jones and Heinemann, 1988; Schmitz et al., 1998; Hamam et al., 2000, 2002; Gloveli et al., 2001). While there is evidence of some hyperpolarization activated inward rectification in the deep layer cells, this rectification is significantly smaller than that of layer II SCs. Along with all superficial principal neurons, deep layer principal neurons also exhibit both monophasic after-spike hyperpolarization potentials (AHPs) and biphasic afterspike depolarization potentials (ADPs) along with AHPs (Alonso and Klink, 1993; Hamam et al., 2000, 2002).

At the network level, the local field potential in EC demonstrates a prominent oscillation in the theta band (4–12 Hz) with a mean frequency of approximately 7 Hz (Mitchell and Ranck, 1980). While the intrinsic oscillatory electrophysiological properties of SCs are suggestive of a local source for theta generation in the EC, which was suggested by Alonso and Llinás (1989), there now seems to be evidence that the theta is generated, or at least initiated, by synaptic input from the MSDB (Jeffery et al., 1995; Zhang et al., 2010; Brandon et al., 2011; Koenig et al., 2011). However, further research is necessary to determine how the intrinsic physiology in EC and feedback projections to the MSDB may help to facilitate or modulate the LFP theta in EC. Indeed, in HCN1 knock-out mice the frequency of the LFP theta in mEC is significantly lower than wild-type, suggesting that intrinsic oscillatory mechanism such as I_h_ have a modulatory effect upon theta (Giocomo et al., 2011). In addition to intrinsic cellular properties in EC, there may also be features of the synaptic architecture which give rise to network level theta, which depend critically upon muscarinic acetylcholine receptor activation (mAChR) (Konopacki et al., 1992).

There are two general classes of receptors that demonstrate an affinity for acetylcholine and can be dissociated according to their binding affinity for either muscarine or nicotine. This review focuses upon modulation in the EC that can be largely attributed to activation of the muscarinic sensitive acetylcholine receptor. mAChRs are G-protein coupled receptors that are expressed in the central nervous system as one of five subtypes in the central nervous system (M1–M5) and can be grouped into two more general classes, M1-like which are associated with the G_*q*_ subtype G-protein coupled receptor and M2-like which are linked with the G_i_ sub-type, (for review see Caulfield and Birdsall, 1998). While mAChR sub-type specific immunostaining has not been done in the EC, mAChR subtypes M1–M4 have been shown to be expressed in the hippocampus (Levey et al., 1995).

## Cholinergic modulation of subthreshold electrophysiology in mEC layer II stellate cells

One way to characterize the range of single cell electrophysiological properties, which is particularly convenient for SCs in mEC, is group them into either subthreshold properties (i.e., electrophysiological phenomenon that occur within a range of membrane potentials that are below firing threshold) and suprathreshold properties (i.e., electrophysiological phenomenon that occur in a range of membrane potentials above the spiking threshold). As mentioned above, mEC SCs show characteristic subthreshold properties including a sag in response to hyperpolarizing current injection, subthreshold membrane potential oscillations, and a resonance frequency that manifests as a difference in amplitude of response to different frequencies of oscillations in input current (Figure 2). These properties are commonly attributed to the hyperpolarization-activated cation current (h current). Recently, it has been shown that I_h_ in SCs is subject to modulation through activation of mAChR (Heys and Hasselmo, 2012). Using voltage clamp, the results of this study demonstrate that cholinergic activation produces a decrease in the amplitude of I_h_ and a hyperpolarizing shift in the activation curve. In addition, I_h_ expressed in more ventrally located SCs display more prominent modulation than I_h_ expressed in SCs located more dorsally in mEC. Heterologous expression of mAChR and h-channels in xenopus oocytes produces a 1.5 to 2-fold slowing of the h-current deactivation time constant, leading to the prediction that the time course of I_h_ deactivation should be similarly affected by cholinergic modulation in SCs (Pian et al., 2007). Yet, surprisingly application of cholinergic agonists does not produce any change in the time course of I_h_ activation or deactivation (Heys and Hasselmo, 2012). Recent work has shown that the co-expression of PEX5R/TRIP8b along with h-channels significantly reduces the influence of cyclic nucleotide and adrenergic activation upon the h-current steady state activation (Zolles et al., 2009). In light of this finding, it is plausible that modulatory effects in SCs are also subject to secondary effects of regulatory subunit expression such as PEX5R/TRIP8b which may alter the effects seen using more simple heterologous expression systems and further research should be conducted to determine whether this is in fact the case.

Related to modulation of I_h_, other characteristic electrophysiological properties of SCs have been shown to be subject to cholinergic modulation. Work from Klink and Alonso (1997) demonstrated that the average frequency of the large amplitude (1–5 mV) subthreshold membrane potential oscillations could be decreased significantly after application of cholinergic agonist, carbachol. Similarly, the frequency and strength of subthreshold membrane potential resonance is reduced after application of carbachol (Figure 2C) (Heys et al., 2010). As such, the voltage clamp work demonstrating cholinergic modulation of I_h_ provides a potential ionic mechanism which could be underlying cholinergic modulation of membrane potential resonance and cholinergic modulation of subthreshold membrane oscillations in SCs (Heys et al., 2010). Furthermore, the results of Heys and Hasselmo (2012) demonstrate that the current generated through expression of the voltage sensitive Kv7 potassium channels (m-current or I_m_) is not expressed in neurons in the EC. This result demonstrates that membrane potential oscillations in EC are not the result of I_m_, which has been suggested in previous research (Yoshida and Alonso, 2007).

Unit recordings from awake-behaving rodents demonstrate that the neurons in the mEC fire in a spatially selective manner. In particular, as the animal explores a 2D environment, a single neuron will fire repeatedly at many selective locations which tile the entire environment and form the vertices of a nearly symmetrical hexagonal grid (Fyhn et al., 2004; Hafting et al., 2005). These “grid cells” have been shown to change in spatial scale systematically along the dorsal-ventral axis of mEC such that grid cells in dorsal mEC have smaller grid fields and have more narrow spacing between the grid fields than grid cells in ventral mEC (Hafting et al., 2005; Brun-Kjelstrup et al., 2008). This finding is particularly interesting when compared with electrophysiological properties of SCs such as I_h_, subthreshold membrane potential oscillation frequency, subthreshold membrane potential resonance frequency, and temporal integration. In each case, the electrophysiological properties change systematically along the dorsal-ventral axis of mEC (Giocomo et al., 2007; Garden et al., 2008; Giocomo and Hasselmo, 2008). Recent work from Giocomo and Colleagues (2011) has demonstrated that knock out of the HCN1 subunit of h-channels in mice produces an expansion of the grid field and the grid field spacing. Similarly, exploration of a novel environment produces an expansion of grid field spacing and grid field size, which returns to baseline upon subsequent explorations of the same environment (Barry et al., 2009). Since there is evidence to suggest that acetylcholine levels in the hippocampus increase during exploration of a novel stimuli (Acquas et al., 1996), the data together suggests the exciting possibility that cholinergic modulation of I_h_ in a novel environment could be underlying the expansion of grid field size and grid field spacing that is observed during exploration of the novel environment (Jeewajee et al., 2008; Barry et al., 2012). In this way, grid expansion in novel environments may orthogonalize inputs to the hippocampus and may cause place field remapping (Barry et al., 2012).

## Cholinergic modulation of suprathreshold physiology in EC

**Figure 3:**
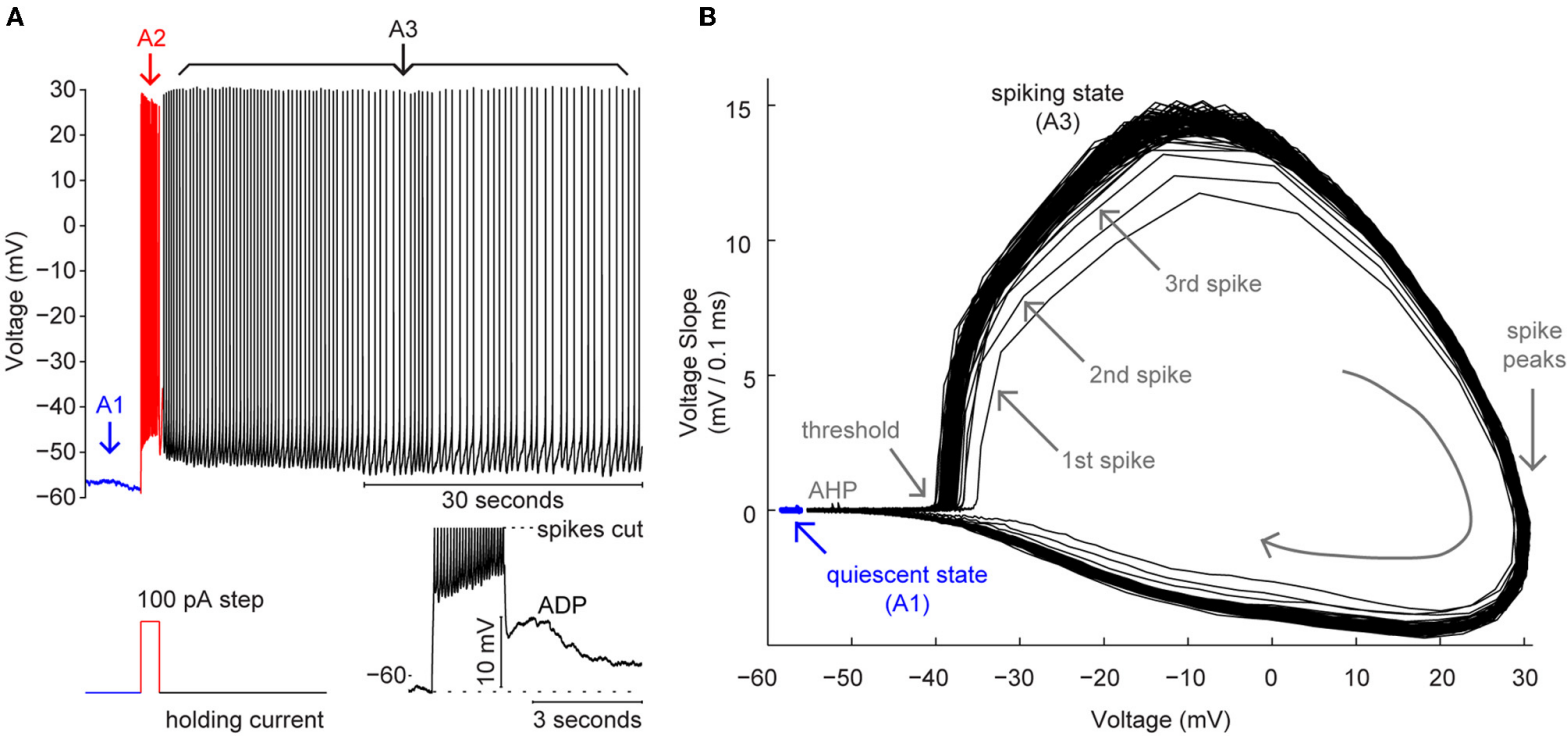
**Bistable persistent spiking. (A) (Top)** Recording from a layer V mEC neuron during the typical protocol used to elicit persistent spiking. Note, the recorded neuron was quiescent (A1) prior to delivery of a 100 pA current step (A2) (stimulus depicted in **Bottom left panel**). After the current step, the recorded neuron spiked continuously (A3, ~1 minute shown). **(A) (Bottom right panel)** Recording from another layer V mEC neuron illustrating a stimulus evoked, after-depolarization. This voltage profile reflects the inward CAN current carried by TRPC channels and often results when the applied holding current (as depicted to the left) is insufficient to support persistent spiking. **(B)** Phase portrait depicting the bistability of the persistent spiking mEC neuron shown in **A (Top)**. Note, in blue, the stable fixed point representing the voltage during quiescence (as in A1). Note also, the spiking state (during persistent spiking) that was achieved following the depolarizing step (as in A3). The spiking trajectory of the first few spikes (indicated with gray arrows) converged quickly to a stable spiking trajectory during persistent spiking. Spike afterhyperpolarizations, spike threshold, and spike peaks are also indicated with grey text and/or arrows. Note lastly, the quiescent and spiking states were exhibited in non-overlapping voltage ranges.

Neurophysiological, functional imaging, and lesion studies in humans and animal models have demonstrated the critical role of cholinergic modulation in working memory (Hasselmo and Stern, 2006) and reveal the central position and function of EC among the prefrontal cortex (PFC) and parahippocampal regions (PHR) recruited by object and place recognition tasks (Otto and Eichenbaum, 1992; Meunier et al., 1993; Eacott et al., 1994; Leonard et al., 1995; Suzuki et al., 1997; Young et al., 1997; Yee and Rawlins, 1998; Schon et al., 2005). (Also see Murray et al., 2000; Hasselmo and Stern, 2006 for related reviews). Systemic disruption of cholinergic modulation impairs performance on working memory tasks including delayed match or non-match to sample tasks (DMS/DNMS), systemic enhancement of cholinergic modulation can improve or rescue performance (Bartus and Johnson, 1976; Bartus, 1978; Penetar and McDonough, 1983; Aigner and Mishkin, 1986; Furey et al., 1997, 2008; Spinelli et al., 2006; Plakke et al., 2008; Myers and Hamilton, 2011), and selective cholinergic deafferentation of EC impairs DNMS performance with novel stimuli (McGaughy et al., 2005). As in PFC, parietal cortex, and throughout PHR (Fuster and Alexander, 1971; Kubota and Niki, 1971; Gnadt and Andersen, 1988; Miyashita and Chang, 1988; Miller et al., 1993, 1996; Chafee and Goldman-Rakic, 1998; Stern et al., 2001; Habeck et al., 2005; Schon et al., 2008), EC neurons exhibit activity selective for aspects of recognition memory for visually presented objects, odors, and locations in DMS/DNMS tasks (Suzuki et al., 1997; Young et al., 1997). In particular, some EC neurons exhibit “delay” activity consisting of elevated spike rates maintained during the interval between sample and test stimulus presentations (Suzuki et al., 1997; Young et al., 1997), providing a potential neural substrate for information held in working memory (Fuster and Alexander, 1971; Kubota and Niki, 1971). In the presence of muscarinic agonists and blockers of synaptic transmission *in vitro*, many EC neurons exhibit intrinsic bistability in that they can be switched to a sustained spiking state from quiescence by a brief depolarizing input. Figure 3A shows an *in vitro* recording made from a synaptically isolated layer V mEC neuron during the typical protocol used to elicit persistent spiking: first, the neuron is held near threshold using tonic applied current (A1); second, a depolarizing current step (2 s, 100 pA) is applied to briefly drive spiking at a high frequency (A2); and last, the applied current is returned to the previous tonic level (A3). As Figure 3A shows, the neuron continues firing (indefinitely) after the step, whereas it had been quiescent prior to the step. These two fundamentally different behaviors were exhibited even though the holding current applied to the neuron was exactly the same before and after the depolarizing step. Thus, as the phase portrait in Figure 3B illustrates, this “bistable” neuron possesses two stable states or behaviors, quiescence, and spiking, with which it can respond to a single, fixed level of input. The spiking state simply reflects the neuron's “memory” of the depolarizing stimulus, suggesting the possibility that the intrinsic mechanisms underlying bistable persistent spiking (PS_B_) *in vitro* could subserve delay activity observed in behaving animals while remembering or “holding” a stimulus in working memory.

Bistable persistent spiking and mechanistically-related, stimulus-evoked afterdepolarizations (Figure 3A, lower right) or plateau potentials have been observed *in vitro* in layers II, III, and V of mEC (Klink and Alonso, 1997; Egorov et al., 2002; Reboreda et al., 2007; Yoshida et al., 2008; Zhang et al., 2011), layer III of lateral EC (Tahvildari et al., 2007, 2008), as well as in most other structures of the extended medial temporal lobe memory system including the hippocampus (El-Hassar et al., 2011), subiculum (Kawasaki and Avoli, 1996; Kawasaki et al., 1999), postsubiculum (Yoshida and Hasselmo, 2009), layers II and III of anterior cingulate cortex (Zhang and Seguela, 2010), lateral amygdala (Egorov et al., 2006), and even primary sensory cortex (Rahman and Berger, 2011). Bistable persistent spiking neurons in layer V (LV) in mEC and basolateral amygdala exhibit an additional feature whereby the firing frequency of persistent spiking can be stably increased, i.e., “graded,” by additional depolarizing or cholinergic inputs, and can be stably decreased by additional hyperpolarizing inputs (Egorov et al., 2002, 2006; Fransen et al., 2006). The multistability of different spike rates exhibited by graded persistent spiking (PS_G_) neurons represents a potential enhancement of working memory mechanisms by allowing information to be held in terms of spike rate rather than only as a function of whether a neuron is spiking or quiescent.

Seminal investigations of persistent spiking identified that the spiking state is dependent on a calcium-sensitive, mixed-cation current (I_NCM_ or I_CAN_) (Klink and Alonso, 1997; Shalinsky et al., 2002). Accumulating evidence now suggests that members of the canonical subfamily of transient receptor potential (TRPCs) membrane channels are responsible for the CAN current that generates persistent spiking (Yan et al., 2009; Zhang and Seguela, 2010; El-Hassar et al., 2011; Wang et al., 2011; Zhang et al., 2011). (For review see Reboreda et al., 2011). TRPC channels are ubiquitously expressed in the nervous system, serving a diversity of functions including calcium regulation and signaling in addition to contributing to the firing properties of neurons (Bollimuntha et al., 2011; Reboreda et al., 2011). TRPC channels also possess several modulatory domains and can be activated by multiple mechanisms involving store-operated calcium or receptor-operated second messenger signaling, depending largely on their heteromeric composition by TRPC1/4/5 or TRPC3/6/7 subunits and organization into microdomains with calcium signaling accessory proteins (Putney, 2005; Ambudkar et al., 2006; Ambudkar and Ong, 2007; Trebak et al., 2007; Pani and Singh, 2009). In brief, bistable persistent spiking of LV mEC neurons and plateau potentials exhibited by cultured cortical neurons appear to depend primarily on the activation of TRPC1/4/5 subunits by the G_α*q*_/PLCβ1 signaling cascade initiated by muscarinic receptor binding (Yan et al., 2009; Zhang et al., 2011), but it is likely that the diversity of CAN current-dependent afterdepolarization, bursting, and persistent spiking phenomena across brain regions results from considerable heterogeneity of mechanisms controlling expression, insertion, and regulation of the TRPC channels.

Bistable persistent spiking (PS_B_) and plateau potentials evoked *in vitro* with muscarinic agonists requires continued receptor binding, since halting stimulation of cholinergic inputs, agonist washout, or subsequent blockade of muscarinic receptors eliminates the persistent response to depolarizing inputs (Kawasaki et al., 1999; Egorov et al., 2006; Tahvildari et al., 2007). Thus, *in vivo* neuronal activity in PHR regions showing PS_B_ would be expected to differ markedly between behavioral states such as navigation, active attending, or remembering which strongly engage the cholinergic system and those that do not.

In addition to muscarinic induction in synaptically isolated neurons, PS_B_ and TRPC-dependent plateau potentials can also be elicited in brain slice preparations using synaptic inputs triggered by stimulation of cholinergic (Egorov et al., 2006) or metabotropic glutamatergic inputs (Egorov et al., 2002; Tahvildari et al., 2007; Yoshida et al., 2008). Importantly, Yoshida and colleagues (2008) showed that bistable persistent spiking (PS_B_) could be elicited by glutamatergic synaptic stimulation even in the presence of muscarinic blockers and ionotropic glutamatergic blockers. This raises the possibility that the metabotropic glutamatergic pathway may converge with the muscarinic pathway to control TRPC channel activation in intact systems, which is critical in light of potentially shared intracellular signaling mechanisms and observations that TRPC activation and persistent spiking can depend on precise control of intracellular calcium (Kinoshita-Kawada et al., 2000; Zhu, 2005; Blair et al., 2009; Zhang et al., 2011) in addition to transmembrane calcium influx (Kawasaki et al., 1999; Egorov et al., 2002; Tahvildari et al., 2008; Zhang and Seguela, 2010). Considerable work remains to be done to elucidate the shared and distinct mechanisms of TRPC activation and regulation in brain regions exhibiting PS_B_.

As described in this section, persistent spiking has now been observed in numerous structures that contribute to working memory. In this context, it is important to distinguish intrinsic persistent spiking (PS_B_, as illustrated in Figure 3, or PS_G_) observed *in vitro* from persistent activity observed in unit recordings from behaving animals (which cannot distinguish intrinsic and network factors). In EC, less than 10% of recorded cells show sample-specific delay activity (Suzuki et al., 1997; Young et al., 1997), whereas much higher proportions of persistent spiking neurons are typically found *in vitro*. This discrepancy could be a consequence of the finite set of stimuli that were used in each *in vivo* experiment, i.e., neurons that did not show persistent activity may have been selective for untested stimuli or stimulus features. However, about half of recorded neurons showing sample-specific delay activity exhibited *decreases* in firing rate, rather than increases, indicating that competitive network mechanisms are likely to shape the spiking output of EC neurons that *in vitro* might otherwise exhibit PS_B_ (Gupta et al., 2012). Recurrent network architectures can also generate persistent activity (Major and Tank, 2004), in which context intrinsic PS_B_ can stabilize working memory representations (Camperi and Wang, 1998). Furthermore, simulations of networks with PS_B_ neurons have reproduced not only delay response activity, but also sample-match suppression and enhancement as in unit recordings during DMS/DNMS tasks (Fransen et al., 2002).

In this section we have introduced the involvement of EC in working memory function assessed with DMS/DNMS tasks, and summarized evidence that the intrinsic mechanisms underlying bistable persistent spiking *in vitro* (Figure 3) are well suited to contribute to persistent activity *in vivo* (Hasselmo and Stern, 2006). We emphasize, however, that intrinsic persistent spiking should not be thought to directly correspond to delay activity in delayed match to sample tasks, since as in all neural systems in intact animals, both cellular and network mechanisms, both of which are subject to modulation, are critical to the behavior of individual neurons and working memory performance in behavioral assays.

## Cholinergic modulation of synaptic transmission

In addition to the influence of acetylcholine on the intrinsic properties of neurons, acetylcholine also modulates synaptic transmission within the EC and hippocampus. The modulation of synaptic transmission may regulate the relative influence of afferent, feedforward excitatory input versus excitatory feedback connections. Computational models suggest that these effects may enhance encoding and attention to external input, while reducing the influence of retrieval and consolidation (Hasselmo et al., 1995; Hasselmo, 1999, 2006) or the top-down influences on perception (Yu and Dayan, 2005).

Early studies in the hippocampus demonstrated that cholinergic stimulation of muscarinic receptors causes presynaptic inhibition of excitatory synaptic potentials in the middle molecular layer of the dentate gyrus (Yamamoto and Kawai, 1967), where synapses from the mEC terminate. In contrast, this presynaptic inhibition is weaker in the outer molecular layer of the dentate gyrus (Kahle and Cotman, 1989), which contains synapses from lEC. Muscarinic presynaptic inhibition was subsequently shown in stratum radiatum of region CA1 of the hippocampus (Hounsgaard, 1978; Valentino and Dingledine, 1981). Acetylcholine suppresses excitatory potentials more in stratum radiatum of region CA1, where CA3 inputs terminate, than in stratum lacunosum-moleculare (SLM), where EC layer III input terminates (Hasselmo and Schnell, 1994). In the piriform cortex, cholinergic modulation causes selective presynaptic inhibition of excitatory feedback potentials in layer Ib, while having a much weaker effect on synaptic potentials in layer Ia receiving afferent input from the olfactory bulb (Hasselmo and Bower, 1992). In region CA3 of hippocampus, muscarinic presynaptic inhibition reduces excitatory transmission at recurrent connections in stratum radiatum (Hasselmo et al., 1995; Vogt and Regehr, 2001), but not at afferent synapses in stratum lucidum (Hasselmo et al., 1995) or stratum lacunosum moleculare (Kremin and Hasselmo, 2007).

In all these regions, the same synapses that show muscarinic presynaptic inhibition of synaptic transmission also show muscarinic enhancement of long-term potentiation in dentate gyrus (Burgard and Sarvey, 1990), piriform cortex (Patil et al., 1998) and region CA1 (Blitzer et al., 1990; Huerta and Lisman, 1995). Stimulation of the medial septum enhances long-term potentiation (LTP) induction *in vivo* (Ovsepian et al., 2004) and scopolamine blocks the LTP enhancement associated with medial septal activity (Leung et al., 2003). Presynaptic inhibition appears to be stronger for synapses with AMPA receptors versus silent synapses in hippocampus (de Sevilla et al., 2002) consistent with physiological evidence that presynaptic inhibition is stronger for recently potentiated synapses in piriform cortex (Linster et al., 2003).

Muscarinic presynaptic inhibition of excitatory synaptic transmission has also been shown in the EC. The presynaptic inhibition in EC suppresses the feedback connections from the subiculum that terminate in the EC (Hamam et al., 2001, 2007) as well as synaptic connections within the EC (Richter et al., 1999). Similar to other structures, muscarinic receptors also enhance induction of long-term potentiation in EC (Cheong et al., 2001). Presynaptic inhibition appears at feedback connections from region CA1 to the subiculum (Kunitake et al., 2004), but also affects input from presubiculum. The effects are not just on feedback, as subiculum also shows selective presynaptic inhibition of medial entorhinal but not lateral entorhinal input. Effects in neocortical structures are overall consistent with this same functional framework, as cholinergic modulation causes presynaptic inhibition of feedback synapses from higher order somatosensory cortex, while having less effect on synaptic potentials elicited in layer IV (Hasselmo and Cekic, 1996). Similarly, acetylcholine suppresses intracortical synaptic potentials but not thalamocortical input in the auditory cortex (Metherate and Hsieh, 2004), and primary visual cortex (Kimura, 2000).

At the same time that feedback is suppressed by muscarinic presynaptic inhibition, activation of nicotinic cholinergic receptors causes enhancement of afferent input to cortical structures. For example, nicotinic enhancement of excitatory synaptic transmission has been shown for the afferent input to hippocampal region CA3 from EC (Giocomo and Hasselmo, 2005) and from the dentate gyrus (Radcliffe et al., 1999), but not for excitatory feedback within CA3. Similarly, in thalamocortical slice preparations of somatosensory cortex (Gil et al., 1997), activation of nicotinic receptors enhances thalamic input but not excitatory feedback synapses. Nicotinic enhancement of glutamatergic transmission has also been shown at the medial dorsal thalamic input to PFC (Gioanni et al., 1999). Nicotinic suppression of GABAergic transmission appears to enhance visual cortex responses to thalamic input (Disney et al., 2007). These effects could enhance the influence of sensory input on cortical spiking activity during encoding, particularly since they would be accompanied by enhancement of the spiking response to afferent input due to muscarinic depolarization of pyramidal cells and reductions in spike frequency accommodation (reviewed in Patil and Hasselmo, 1999; Hasselmo and McGaughy, 2004).

Computational modeling shows that these cholinergic effects on synaptic transmission may enhance attention and encoding of stimuli in the environment (Hasselmo and Schnell, 1994; Hasselmo et al., 1995; Hasselmo, 2006). The depolarization of neurons by muscarinic receptors coupled with nicotinic enhancement of afferent transmission will ensure a stronger response to afferent input. At the same time, the muscarinic presynaptic inhibition of excitatory feedback prevents interference from the retrieval or consolidation of previously formed memories (Hasselmo et al., 1995; Hasselmo, 1999, 2006) and reduces top-down influences on perception (Yu and Dayan, 2005). Interference is specifically prevented during the encoding of new stimuli because the selective muscarinic presynaptic inhibition of excitatory feedback occurs during muscarininc enhancement of LTP at the same synapses (Hasselmo et al., 1995; Hasselmo, 2006). In this manner, the effects of acetylcholine may work together to enhance attention to external stimuli, and enhance encoding through an increase in synaptic modification coupled with a reduction of interference from previous memories.

## Cholinergic modulation of interneurons

Despite the fact that interneurons only comprise about 10% of the cortical neuronal population (Rudy et al., 2011), interneurons make up an extremely diverse group of cells. Accordingly, the classification of interneurons has proven difficult. To date the most complete attempt has been provided in the hippocampus by Freund and Buzsáki (1996), and the reader is referred there for a comprehensive review. Here only a brief introduction to interneuron classes within the hippocampus (CA1 and CA3) will be provided, with a focus on correlations between expression of different neurochemicals and axonal targets onto principal cell spatial domains. This introduction is by no means a complete description of all interneurons. It is merely meant to provide a basic background for readers who may not be familiar with different interneuron subtypes that will be discussed throughout this section.

The calcium-binding protein parvalbumin (PV) is expressed in fast spiking basket and chandelier cells of the hippocampus (Kawaguchi et al., 1987; Kosaka et al., 1987) and neocortex (Kawaguchi and Kubota, 1997). Basket cell axons surround the soma and proximal dendrites, whereas axons of chandelier cells synapse on axon initial segments of principal cells (Sik et al., 1995; Buhl et al., 1994). Some basket cells lack PV and instead express the neuropeptide(s) cholesystokinin (CCK) and/or vasoactive intestinal polypeptide (VIP, Kosaka et al., 1985; Freund and Katona, 2007). CCK basket cells can be differentiated physiologically from PV basket cells by their regular spiking phenotype (Cea-del Rio et al., 2010; Szabó et al., 2010). Recently, in CA1, a second class off CCK expressing interneurons have been described as containing axons targeting principal cell dendrites within the stratum radiatum (Klausberger et al., 2005; Cea-del Rio et al., 2011) and therefore are sometimes referred to as Schaffer-collateral associated cells.

Immunostaining for somatostatin (SOM) has revealed extensive labeling in all regions of the hippocampus (Köhler and Chan-Palay, 1982; Somogyi et al., 1984; Sloviter and Nilaver, 1987). Within CA1, SOM expressing interneurons are largely located in the stratum oriens/alveus (Gulyás et al., 2003), and can be further separated into subtypes based upon their synaptic targets. The first group is termed O-LM cells because they have cell bodies and dendrites confined to stratum oriens, and axons that extend to the lacunosum moleculare, where they give off extensive axon collaterals (Katona et al., 1999a). The second group sends axons to CA1 and CA3 and is therefore termed back-projection cells (Sik et al., 1995). The last group projects to the medial septum and is therefore referred to as hippocampal-septal interneurons (Gulyás et al., 1996).

Lastly, there are interneurons in both the hippocampus and neocortex that selectively target other interneurons. VIP expressing interneurons in the hippocampus synapse on SOM O-LM, calbindin, as well as other VIP interneurons (Acsády et al., 1996). Interneurons expressing the calcium binding protein calretinin form dense dendro- and axo-dendritic connections between one another and target other interneurons including calbindin, VIP/CCK basket, and SOM O-LM cells (Gulyás et al., 1996). Similarly, the neocortex contains VIP/calretinin double bouquet and arcade cells that also target other interneurons (Kawaguchi and Kubota, 1997).

As suggested above, most of what we know about interneurons has come from research conducted in the hippocampus and neocortex. Surprisingly, data from interneurons of the EC is for the most part lacking, despite the structure's critical role in gating information flow between the neocortex and hippocampus. However, recent advances in genetics have led to the production of transgenic mouse lines expressing enhanced green fluorescent protein (EGFP) in specific populations of interneurons. These transgenic strains have provided powerful tools to investigate neuromodulation in specific and easily identifiable interneurons. Investigations with these strains have been focused in the hippocampus and neocortex, while very little work has been reported from the EC. Therefore, in the remainder of this section, data on the cholinergic modulation of interneurons from the hippocampus (CA1 and CA3 subfields), neocortex, and, when available, the EC will be briefly reviewed with the goal of raising important questions to guide future experiments in interneurons of the EC.

Application of mAChR agonists by bath (carbachol, muscarine, ACh) or focal (ACh) perfusion induces a number of membrane potential responses in CA1 interneurons. These responses predominantly include a sustained depolarization, but also hyperpolarization, biphasic (hyperpolarization, followed by depolarization), and to a lesser extent oscillatory and unresponsive cells (McQuiston and Madison, 1999a). These diverse responses have also been reported upon endogenous ACh release via electrical stimulation in CA1 stratum oriens (Widmer et al., 2006). Additionally, pharmacological or synaptic muscarinic activation of CA1 interneurons transforms responses to intracellular current injection from afterhyperpolarizations (AHP) to afterdepolarizations (ADP) (McQuiston and Madison, 1999b), increasing cell excitability. Similar results have been found in the neocortex. For instance, in the frontal cortex, application of muscarine or carbachol to CCK interneurons results in a depolarizing or biphasic response, whereas in SOM and VIP expressing interneurons only depolarizing responses were reported (Kawaguchi, 1997). In contrast, PV basket cells of the frontal cortex are unresponsive to muscarinic activation (Kawaguchi, 1997). Given muscarinic responses are diverse but conserved in interneurons of the hippocampus and neocortex, it is likely that the EC will show similar responses across interneuron subtypes.

Recent studies utilizing EGFP expressed in CCK or PV interneurons of CA1 have provided significant insight to the studies above. For instance, the transformation from an AHP to an ADP, as well as the biphasic membrane potential response, requires activation of M1 and M3 mAChRs. These responses were found in CCK basket and Schaffer-collateral associated cells (Cea-del Rio et al., 2010, 2011), as well as in SOM expressing O-LM interneurons (Lawrence et al., 2006a). This latter study showed the emergence of the ADP was dependent on inhibition of the M-current and a slow calcium-activated potassium channel, as well as activation of calcium-dependent non-selective cationic current (ICAN). In contrast, the membrane potential depolarization and increased firing frequency in CA1 PV basket cells require activation of M1 mAChRs (Cea-del Rio et al., 2010, 2011).

Immunocytochemical studies have shown differential expression of muscarinic receptor subtypes within the whole brain (Levey et al., 1991) and hippocampus (Levey et al., 1995; Hájos et al., 1998). Most notably, in the hippocampus M2 mAChRs are densely expressed on axon terminals of PV basket and axo-axonic cells, while in dendritic targeting interneurons (calretinin and SOM) M2 mAChRs are expressed on the soma and dendrites (Hájos et al., 1998). Similarly, M2 mAChRs are expressed on PV basket cells of the auditory cortex (Salgado et al., 2007) and EC (Chaudhuri et al., 2005). Activation of the M2 mAChRs has been shown to decrease the amplitude of unitary IPSPs in CA3 pyramidal cells (Szabó et al., 2010), IPSCs in pyramidal cells of the auditory cortex (Salgado et al., 2007) and IPSPs in pyramidal and SCs of the EC (Apergis-Schoute et al., 2007). These data strongly support a heterosynaptic regulatory role for M2 mAChRs, where their activation on interneurons inhibits the synaptic release of GABA and decreases inhibitory potentials in pyramidal cells. In contrast, M1 mAChRs and M3 mAChRs activation have a predominantly excitatory effect on interneurons, increasing levels of inhibition in principal cells. Interesting questions remain unanswered within the EC. For instance, in what interneurons are muscarinic receptors other than M2 expressed, and are they localized in particular cellular compartments? Can muscarinic receptor expression be correlated with interneuron innervation of particular principal cell domains, and do the same neurochemical markers (CCK, PV, SOM, neuropeptide Y (NPY), calbindin, calretinin, etc.) correlate with similar innervation of principal cell domains as seen in the hippocampus (reviewed by Freund and Buzsáki, 1996)?

Van der Zee and colleagues have contributed extensively to mAChR immunocytochemistry of the hippocampus, neocortex, and amygdala (reviewed in Van der Zee and Luiten, 1999). In the interest of space, here we will focus on their data concerning the hippocampus. Their body of work uses M35, a pan-mAChR antibody which labels all muscarinic receptors that are in an activated state (André et al., 1984). Therefore, M35 immunoreactivity (ir) allows visualization of phosphorylated/internalilzed mAChRs and can be used as a tool to investigate the functional cholinergic properties of a cell or network. Immuncytochemical studies in naïve animals have found M35-ir in basket cells within stratum pyramidale of CA1-CA3 as well as other interneuron types in SLM and stratum oriens/alveus (Van der Zee et al., 1989, 1991a). Similar studies found a high degree of colocalization between M35 and the GABA markers PV and SOM (Van der Zee et al., 1991a,b, 1993). Out of 2730 hippocampal interneurons expressing mAChRs, 33% colocalized with SOM, 52% with PV, and 72.8% with SOM and/or PV. Furthermore, 97.5% of hippocampal GAD positive cells express mAChRs, suggesting a ubiquitous role of cholinergic modulation in interneurons.

The use of M35 as a functional tool has been shown by significant changes in hippocampal M35-ir before and after learning in various paradigms. For example, in the holeboard task, a spatial learning task where rats learn to find food rewards arranged in fixed patterns according to the location of external cues, trained rats show dense M35-ir in the cell soma and dendrites of hippocampal pyramidal cells. This is in contrast to naïve and pseudo-trained (all holes baited with food) animals who show M35-ir in interneurons (Van der Zee et al., 1995). Similarly, in eye blink conditioning, an associative learning task, hippocampal M35-ir shifts from being high in interneurons of naïve rabbits, to being high in pyramidal cells of conditioned rabbits (Van der Zee et al., 1997). The data above suggests that a shift in muscarininc activity occurs in the hippocampus during learning. Before learning mAChRs activity is high in interneurons, while after learning, mAChR activity is high in principal cells.

The data describing a shift in mAChR activity during plasticity correlate well with the computational model by Hasselmo et al. (1995) describing acetylcholine dynamics during encoding and retrieval. The model utilizes experimental data describing differential effects of pre- and postsynaptic mAChR activation in hippocampus and piriform cortex (Hasselmo and Bower, 1992; Hasselmo and Schnell, 1994; Hasselmo et al., 1995). High acetylcholine levels support encoding of new information by silencing intrinsic (local) input via activation of presynaptic mAChRs and at the same time enhancing extrinsic input via activation of postsynaptic mAChRs. Postsynaptic activation causes depolarization, which increases LTP and facilitates learning. In contrast, low acetylcholine levels favor retrieval because in the absence of presynaptic inhibition and postsynaptic depolarization, afferent activity dominates. Data on M35-ir can be applied to this model as follows: before learning M35-ir is initially low in pyramidal cells, indicating mAChRs are unbound and open for encoding. After training, M35-ir is very high indicating mAChRs have been activated and internalized. In this situation the network would be in recall mode. Given the predominant excitatory nature of mAChR activation, in non-learning (low acetylcholine) conditions, mAChR activation on interneurons could help to keep pyramidal cell excitability low and maintain an open window for encoding. Whether similar patterns of M35-ir are present in the EC before and after learning is unknown. However, given the role of the EC in spatial memory and gating information between the hippocampus and neocortex, it seems reasonable that similarities would exist.

Recent studies using the 5HT3aR-BAC EGFP transgenic mouse line have reported broad expression of the ionotropic serotonin (5HT3a) receptor in the superficial layers of the primary somatosensory cortex (Lee et al., 2010; Rudy et al., 2011). This expression pattern is consistent with immunocytochemical studies showing diffuse expression, likely indicative of localization on interneurons (Tecott et al., 1993). Accordingly, 5HT3aRs are expressed in all interneurons originating from the caudal ganglionic eminences (including VIP, NPY, and CCK expressing interneurons) but are absent in PV and SOM interneurons (Lee et al., 2010). Moreover all 5HT3aR expressing interneurons co-express nicotinic AChRs (nAChRs). The physiological effect of both serotonergic and nicotinic activation is a fast depolarization. Focal somatic puffs of 100 μM mCPBG or 1 mM carbachol elicit a burst of spikes, while 100 μM nicotine elicits depolarizations (Lee et al., 2010). Very similar results have also been reported in an earlier study by Ferezou et al. (2002). McQuiston and Madison (1999c) have shown in CA1 interneurons of stratum radiatum/stratum lacunosum moleculare that fast nAChR depolarization is mediated by alpha-7 subunits, while interneurons in stratum oriens, display a dual fast and non-alpha-7 mediated slow depolarization. Given the excitatory nature of both serotonergic and nicotinic responses, a portion of these EGFP interneurons are likely to take part in feedforward inhibition of principal cells. In fact work has been done within the EC showing that activation of 5HT3aRs decreases acetylcholine release, through apparent inhibition of principal cells (Ramírez et al., 1996). In support of this, focal iontophoretic activation of nAChRs on CA1 PV basket cells releases quantal GABA. This release is independent of action potentials but dependent on the activation of Cav3.1 T-type calcium channels and calcium from internal stores (Tang et al., 2011). In contrast to feedforward inhibition, VIP cells in somatosensory cortex (Dávid et al., 2007) and the hippocampus (Acsády et al., 1996) have been shown to selectively synapse onto other interneurons. Therefore a subset of EGFP (VIP/5HT3aR/nAChR) interneurons could play a role in disinhibiting principal cells.

Indeed a form of disinhibition, termed depolarization-induced suppression of inhibition (DSI), exists between CCK expressing basket cells and principal cells of the neocortex (Fortin et al., 2004; Trettel et al., 2004) and hippocampus (Katona et al., 1999b). CCK interneurons express cannabinoid-1 receptors (CB1R) on their nerve terminals (Tsou et al., 1999; Hájos et al., 2000), which bind retrograde endocannabinoids released from principal cells upon depolarization (Ohno-Shosaku et al., 2001). CB1R activation then inhibits GABA release in CCK interneurons (Katona et al., 1999b), thereby suppressing IPSCs in principal cells (Hoffman and Lupica, 2000). Carbachol enhances DSI in the hippocampus (Kim et al., 2002; Ohno-Shosaku et al., 2003) and this enhancement is due to activation of M1 and M3 mAChRs (Ohno-Shosaku et al., 2003) and subsequent modulation of G-proteins (Kim et al., 2002). In the EC, CCK interneurons selectively synapse on principal cells expressing calbindin (Varga et al., 2010). These cells are likely SCs and they target the contralateral EC, as opposed to those forming the perforant path. This selective targeting of CCK cells suggests a specialized function, perhaps utilizing DSI as a mechanism of action.

This specialized function may well include the synchronization of the entorhinal cortices across hemispheres, as there is convincing evidence that basket cells (PV and CCK) play critical roles in synchronizing oscillatory activity in principal cells (reviewed by Buzsáki, 2002; Whittington and Traub, 2003; Klausberger et al., 2005; Freund and Katona, 2007). Cobb et al. (1995) have shown that single basket cells of CA1 can entrain subthreshold membrane potential oscillations and spiking of multiple pyramidal cells. Interestingly the phase of interneuron and pyramidal activity is separated by 180°, similar to phase differences between CA1 perisomatic interneurons and pyramidal cell firing *in vivo* (Klausberger and Somogyi, 2008). Moreover, CB1R activation has been shown to interrupt kainate-induced gamma activity in the hippocampus (Hájos et al., 2000) and EC (Morgan et al., 2008). Gamma oscillations are also induced by bath application of carbachol in all layers of somatosensory cortex (Buhl et al., 1998) and in CA3 where it is then transferred to CA1 likely through excitatory Schaffer-collateral afferents (Fisahn et al., 1998). In both regions, GABA_A_ receptor and AMPA/kainite receptor activation are necessary for the emergence of gamma activity. In addition, electrical synapses in PV basket cells of the neocortex (Gibson et al., 1999; Tamás et al., 2000; Blatow et al., 2003; Whittington and Traub, 2003) synchronize large numbers of interneurons and principal cells and are crucial for gamma activity. Additionally, mAChR activation induces theta rhythmic IPSPs in hippocampal pyramid cells which are disrupted by subsequent activation of CB1Rs (Reich et al., 2005). This mAChR activation is physiologically relevant as optogenetic stimulation of cholinergic fibers of the MSDB activates CCK interneurons resulting in theta rhythmic IPSC bursts in CA1 pyramidal cells. Moreover this rhythmic inhibition is abolished with postsynaptic depolarization and this DSI is blocked with application of a CB1R antagonist (Nagode et al., 2011).

Freund (2003) has suggested that CCK interneurons transmit information of the emotional state (mood) of the animal and act to fine-tune principal cell activity, while PV basket cells control the clock-like rhythm of principal cells. In agreement with this, the data above suggests that CCK interneurons, via CB1Rs, modulate, while PV basket and principal cells generate and maintain oscillatory activity. Additional support for a modulatory role in oscillatory activity by CCK interneurons is provided by the rapid desensitization of nAChRs (McQuiston and Madison, 1999c) and 5HT3aRs (Sugita et al., 1992). Rapid desensitization results in transient depolarization following receptor activation. For this reason it has been suggested that nicotinic receptors are unlikely to make significant contributions to the generation of theta (Buhler and Dunwiddie, 2001), and it seems reasonable to suppose the same for 5HT3Rs. This transient nature of nAChR and 5HT3aR activation seems well posed to function as a switch between oscillatory states, or oscillatory and non-oscillatory states. Accordingly, Vertes and Kocsis (1997) have suggested a role for serotonin in the desynchronization of the hippocampal theta rhythm, and nAChR activation in the hippocampus has been shown to switch a purely mAChR induced oscillatory bursting mode, characterized by individual depolarizing events occurring at low frequencies (<1 Hz), to a theta-mode discharge, characterized by rhythmic oscillatory depolarization between 4 and 12 Hz (Cobb et al., 1999).

It is likely that the modulatory functions of CCK expressing cells are mirrored in the EC as the median raphe nucleus sends serotonergic projections to both the hippocampus and EC (Vertes et al., 1999), and 5HT3aR immunoreactivity is found throughout the hippocampal formation (Morales et al., 1998; Miquel et al., 2002) and the EC (Miquel et al., 2002). In addition, both the hippocampus (Nyakas et al., 1987) and EC (Alonso and Köhler, 1984) receive cholinergic input from the MSDB. Moreover, theta activity of deep layers of the EC is in phase with CA1, while superficial layers are in phase with dentate gyrus (Mitchell and Ranck, 1980; Alonso and Garcia-Austt, 1987), suggesting oscillatory activity of the two regions are similarly governed according to synaptic connectivity between the two regions. To date, direct investigations of the physiological properties of CCK cells in the EC have not been conducted. Given that entorhinal SCs display intrinsic subthreshold oscillations with depolarization (Alonso and Llinás, 1989; Klink and Alonso, 1993) and carbachol induces depolarization and subthreshold oscillations at rest, future work focusing on the effects of nicotinic, muscarinic, serotonergic, and endocannabinoid activation in CCK interneurons can provide valuable information on possible modulation of these oscillatory properties in the EC.

Basket cells are not the only interneurons that can regulate the phase of pyramidal cell activity. Interneurons of the stratum lacunosum moleculare display oscillatory behavior in response to muscarinic activation by carbachol (Chapman and Lacaille, 1999). Moreover minimal stimulation at 3 Hz causes rhythmic rebound spikes in pyramidal cells that are ~180° out of phase with the activation of SLM interneurons. Perisomatic interneurons have powerful control over cell output, while dendritic interneurons control integration of inputs and synaptic plasticity (Miles et al., 1996). An important question pertaining to the EC is what cell types mediate control over dendritic integration? Some insight can be gained through comparison of data from SOM expressing interneurons of the hippocampus and neocortex. The transgenic GFP-expressing inhibitory neuron (GIN) mouse line selectively expresses EGFP in SOM interneurons (Oliva Jr. et al., 2000). In the neocortex of GIN mice, Martinotti cells are found in layers II, III, V, and VI and their axons extend into superficial layers and give off dense axonal collaterals in layer I (McGarry et al., 2010). These same morphological results were found by Kawaguchi and Kubota (1996) in rats. In addition, McGarry et al. (2010) found two other types of SOM cells that contained axons that did not reach layer I and lacked dense collaterals. It was suggested that these cells could innervate distant regions of cortex or contained immature processes. SOM neurons of the hippocampus include O-LM cells, which are very similar to Martinotti cells in that their soma and dendrites are located in the region of principal cell axons (stratum oriens) and their axons extend to lacunosum moleculare where they too have diffuse axonal collaterals (Katona et al., 1999a). Additionally, hippocampal-septal interneurons, a subset of CA1 SOM interneurons, project to the medial septum, CA1 and CA3, where they selectively target other interneurons (Gulyás et al., 2003). The question remains if similar morphological properties exist in the EC? It is known that SOM interneurons are present in the layers II, III, and V of the EC, but the majority of SOM expressing cells are non-GABAergic (Wouterlood and Pothuizen, 2000). Therefore, future studies focusing on possible morphological differences between GABA positive and GABA negative SOM cells are warranted.

The conserved morphology and function of SOM cells of the neocortex and hippocampus suggests that these cells play a general role in feedback inhibition across the brain. In fact, in layer V somatosensory cortex, SOM late spiking Martinotti cells take part in disynaptic inhibition of pyramidal cells (Silberberg and Markram, 2007). This study reported that Martinotti cells contacted 79% of neighboring pyramidal cells, while 68% of pyramidal cells contacted neighboring Martinotti cells. This high degree of local connectivity is also present in layer II/III of the frontal cortex, where 70% of Martinotti cells within 200 μm of pyramidal cells are connected (Fino and Yuste, 2011). This high degree of connectivity between Martinotti and pyramidal cells results in supralinear inhibition of pyramidal cells. In layer II/III of somatosensory cortex, spiking of two pyramidal cells increases inhibition in a third pyramidal cell in a supralinear manner. This supralinear feedback inhibition upon activation of two pyramidal cells is due to a tenfold increase in the recruitment of SOM interneurons compared to when only a single pyramidal cell is firing (Kapfer et al., 2007).

In addition to feedback inhibition, Martinotti cells also synchronize their activity in response to mAChR or mGluR activation (Beierlein et al., 2000; Fanselow et al., 2008). Cholinergic-induced synchronization is in the theta range (Fanselow et al., 2008) and is dependent on the activation of M1 and M4 mAChRs (Beierlein et al., 2000). Moreover, both studies show that synchronization is independent of chemical synapses as it is TTX resistant. Moreover, Martinotti cell synchronization via gap junctions also synchronizes activity in regular spiking as well as fast spiking cells. As mentioned above, muscarinic activation in hippocampal O-LM cells increases excitability and transforms an AHP to an ADP, all of which are mediated by M1 and M3 mAChRs (Lawrence et al., 2006a). In addition, mAChR activation increases spike reliability to sinusoidal inputs increasing the bandwidth at which O-LM cells can maintain firing on all cycles from 5–12 Hz to 7–17 Hz (Lawrence et al., 2006b). This increase in the theta bandwidth is due to the increased slope during the upswing, which can be attributed to the ADP. O-LM cells densely innervate pyramidal cells and therefore muscarinic activation can potentially synchronize large populations of both interneurons and pyramidal cells to help coordinate theta rhythmic activity in the cortex. Given that the EC displays a prominent theta rhythm, and the largest amplitude theta rhythm of CA1 is in lacunosum moleculare where entorhinal afferents synapse (Buzsáki, 2002), it seems likely that SOM interneurons could contribute to the generation of theta in the EC as well as in the neocortex and hippocampus.

Both O-LM (Maccaferri and McBain, 1996) and Martinotti cells (Wang et al., 2004) express the h-current which paces action potential firing and contributes to AHP in O-LM cells. Muscarinic activation has been shown to modulate the time constant of the h-current (Pian et al., 2006, 2007) and muscarinic activation decreases subthreshold resonance (Heys et al., 2010) and induces subthreshold membrane potential oscillations (Klink and Alonso, 1997) in layer II EC SCs. Both subthreshold membrane potential resonance (Shay et al., 2012) and subthreshold membrane potential oscillations (Dickson et al., 2000a,b) have been associated with expression of I_h_. It still remains to be seen whether SOM expressing interneurons in the EC possess I_h_ or if they display similar subthreshold resonance properties as reported in the hippocampus (Pike et al., 2000; Zemankovics et al., 2010). If resonance is found within entorhinal SOM interneurons, an important question to ask is whether similar frequency gradients are observed as in stelllate cells (Giocomo et al., 2007)? If so, entorhinal SOM interneurons could function to synchronize and bind SCs at specific frequencies along the dorsal-ventral axis. This could have implications for the generation of grid cells as gradients in their field size and spacing have been observed along the dorsal-ventral axis of mEC (Fyhn et al., 2004; Sargolini et al., 2006; Brun-Kjelstrup et al., 2008). Lastly, can subthreshold oscillations and resonance properties be modulated by acetylcholine and in what ways? Assuming GIN mice express detectable amounts of EGFP in SOM interneurons of the EC, this transgenic line could prove valuable to answering these questions.

To summarize, the activation of muscarinic receptors has differential consequences on membrane potential, depending on which subtypes are expressed on a given cell or cell compartment. These diverse responses could play various roles in information processing. For example, muscarinic induced membrane potential hyperpolarization could function to bring a cell further from threshold, while the ADP could then selectively enhance inputs with sufficient strength to reach threshold, thus enhancing the signal to noise ratio (McQuiston and Madison, 1999b). In addition, a muscarinic induced membrane potential depolarization and ADP could sufficiently increase firing frequency to release neuropeptides such as SOM, CCK, VIP, and NPY, which by themselves have diverse effects. Additionally, muscarinic activation induces synchronized oscillatory activity in both interneurons and pyramidal cells, which could contribute to the generation of theta and gamma rhythms. On the other hand muscarinic activation also disrupts rhythmic inhibition of principal cells through retrograde activation of endocannabinoid receptors on CCK interneurons. The activation of ionotropic nicotinic AChRs and 5HT3aRs, also expressed by CCK interneurons, are also likely to modulate oscillatory states, suggesting cholinergic modulation can play dual roles in the generation of rhythms. Lastly, cholinergic activation affects intrinsic properties including subthreshold oscillations and resonance, which if displayed by entorhinal interneurons could have functional implications for grid cell function.

## Summary

In this review, we have described multiple effects of acetylcholine on the physiology of entorhinal neurons. Cholinergic activation of muscarinic receptors causes a decrease in the resonance frequency of SCs in layer II of EC that appears to arise from cholinergic decreases in the hyperpolarization activated cation current. Acetylcholine also activates a calcium-sensitive non-specific cation current that enhances the appearance of persistent spiking in entorhinal pyramidal cells. In addition, muscarinic receptors cause presynaptic inhibition excitatory synaptic transmission at feedback connections to the EC, and at a subset of output synapses from mEC to the middle molecular layer of the dentate gyrus. Modeling has shown how these modulatory effects of acetylcholine could alter the dynamics of EC in a manner that could contribute to the role of cholinergic modulation in spatial representations for episodic memory function (Fransen et al., 2002, 2006; Hasselmo and Stern, 2006; Hasselmo, 2008, 2012). These clear effects of cholinergic modulation motivate future studies to demonstrate how the currents underlying persistent spiking could influence other dynamical properties of entorhinal neurons, as well as to determine whether acetylcholine influences entorhinal inhibitory interneurons as strongly as it influences the oscillatory and repetitive spiking properties of hippocampal interneurons. Further analysis of cellular mechanisms combined with computational modeling will help us understand how the dense cholinergic innervation of EC alters cellular dynamics to underlie the important role of cortical acetylcholine in cognitive function.

### Conflict of interest statement

The authors declare that the research was conducted in the absence of any commercial or financial relationships that could be construed as a potential conflict of interest.
